# Southern African HIV Clinicians Society Guidance on the use of dolutegravir in first-line antiretroviral therapy

**DOI:** 10.4102/sajhivmed.v19i1.917

**Published:** 2018-10-17

**Authors:** Michelle A. Moorhouse, Sergio Carmona, Natasha Davies, Sipho Dlamini, Cloete van Vuuren, Thandekile Manzini, Moeketsi Mathe, Yunus Moosa, Jennifer Nash, Jeremy Nel, Yoliswa Pakade, Joana Woods, Gert van Zyl, Francesca Conradie, Francois Venter, Graeme Meintjes

**Affiliations:** 1 Wits Reproductive Health and HIV Institute, Faculty of Health Sciences, University of the Witwatersrand, South Africa; 2 National Health Laboratory Services, South Africa; 3 Department of Medicine, University of Cape Town, South Africa; 4 Southern African HIV Clinicians Society, South Africa; 5 Private Practice, Vereeniging, South Africa; 6 Department of Infectious Diseases, University of KwaZulu-Natal, South Africa; 7 Department of Medicine and Institute of Infectious Disease and Molecular Medicine, University of Cape Town, South Africa

The Southern African HIV Clinicians Society would like to update all HIV-treating clinicians with regard to the use of dolutegravir in women of childbearing potential (WOCP).

In preliminary data from the Tsepamo study in Botswana, it was found that 0.94% (95% confidence interval [CI]: 0.37 – 2.4) of babies (4/426) born to women who were taking dolutegravir periconception had neural tube defects (NTDs), compared with 0.1% of babies (14/11 173) of women taking other antiretroviral drugs (ARVs) in the periconception period.^1^ No NTDs were observed in pregnancies where dolutegravir was initiated later in pregnancy. Further data from the Tsepamo study were presented at AIDS 2018: the updated number of NTDs with periconception dolutegravir exposure in the Tsepamo cohort is 4/596, 0.67% (95% CI: 0.26 to 1.7). The next formal analysis will occur after 31 March 2019 and will include women exposed to dolutegravir from conception before the recent change in guidance. Tsepamo plans to expand the number of study sites, increasing the coverage from 45% to 72% of births in Botswana with a projected denominator of over 1200 by March 2019.^1^

Prior to the data from Botswana, reproductive toxicology studies had not shown any concerning findings. To date, other data on the use of dolutegravir in pregnancy, including data from the Antiretroviral Pregnancy Registry, clinical trials and post-marketing surveillance, have not indicated a risk of NTDs.

The World Health Organization (WHO) launched new interim guidance on HIV treatment at AIDS 2018, recommending dolutegravir for everyone aged six years and above. Based on limited data, WHO notes that there are safety concerns regarding the use of dolutegravir periconception.^2^

World Health Organization recommendations for WOCP include the following:

- Dolutegravir-based first-line antiretroviral therapy (ART) is recommended for:
  ▪ all pregnant cent girls^2,3^
  ▪ women and adolescent girls with effective contraception or not of childbearing potential.
- Women and adolescent girls of childbearing potential who want to become pregnant or have no effective contraception should use efavirenz-based (600 mg) first-line ART.^2,3^
- Consider the balance of benefits and risks, including fertility levels, contraceptive availability and coverage, pretreatment resistance to non-nucleoside reverse transcriptase inhibitors at the population level, drug availability and the maternal and infant toxicity profile when selecting the optimal ARV drug regimen for WOCP.^2,3^
- Strengthen the integration of sexual and reproductive health services within HIV treatment programmes to ensure reliable and consistent access to contraception for women and adolescent girls living with HIV.^2,3^
- A woman-centred approach should be adopted: healthcare providers should provide women with information and options to allow for informed choices about using lifelong ART regimens.^2,3^

## Southern African HIV Clinicians Society guidance

- A woman-centred approach should be adopted: healthcare providers should give women information and options to allow for informed choices about using lifelong ART regimens.
- Dolutegravir-based first-line ART is recommended for:
  ▪ all pregnant (from eight weeks after conception) and breastfeeding women and adolescent girls
  ▪ women and adolescent girls on effective contraception or not of childbearing potential.
- Women and adolescent girls of childbearing potential who want to become pregnant or have no effective contraception should be adequately counselled about the potential risks and benefits of dolutegravir- versus efavirenz-based ART and should be offered the choice of both treatments. This discussion should be documented, preferably along with consent from those women opting for dolutegravir-based ART.
- If pregnancy is confirmed in the first eight weeks while a woman is taking dolutegravir, she should be adequately counselled about the potential risks and benefits of dolutegravir- versus efavirenz-based ART and should offered the choice of both treatments. This discussion should be documented, preferably along with consent from those women opting for dolutegravir-based ART. The risk and benefits of switching during pregnancy should also be discussed. Switching is associated with a small risk of viraemia in a previously virologically suppressed patient, which may result in risk of mother-to-child transmission and resistance.

While this is an early signal, it warrants careful pharmacovigilance and further evaluation. As more information becomes available, we will update our guidance.
